# The Influence of a Vitrectomy on the Diurnal Intraocular Pressure

**DOI:** 10.1155/2015/427808

**Published:** 2015-06-15

**Authors:** Yong Woo Lee, Joon Mo Kim, Seong Hee Shim, Da Yeong Kim, Jeong Hun Bae, Ki Ho Park

**Affiliations:** ^1^Department of Ophthalmology, Kangbuk Samsung Hospital, Sungkyunkwan University School of Medicine, Seoul 110-746, Republic of Korea; ^2^Department of Ophthalmology, Seoul National University Hospital, Seoul National University College of Medicine, Seoul, Republic of Korea

## Abstract

*Purpose*. To evaluate the diurnal intraocular pressure (IOP) in eyes after vitrectomy compared to that of healthy eyes.* Methods*. Twenty-one patients who had undergone vitrectomy and 21 age- and gender-matched normal controls were enrolled during the same period. We measured the diurnal IOP every two hours between 9 a.m. and 11 p.m. in all patients who were admitted for cataract surgery. Patients with a history of eye surgery (not including vitrectomy) or use of a medication that is associated with IOP were excluded. The IOP and ocular parameters of patients were compared with the same patients' fellow healthy eyes and with normal eyes of age- and gender-matched controls.* Results*. There were no significant differences between vitrectomized eyes and normal fellow eyes with regard to all IOP parameters including the maximum, minimum, and IOP fluctuation values. Diurnal fluctuation of IOP (or the difference between the maximum and minimum IOP) was larger in vitrectomized eyes than it was in age- and gender-matched control eyes.* Conclusions*. Vitrectomy did not markedly affect the IOP. Although there were no severe complications after vitrectomy, the IOP fluctuation was wider in vitrectomized eyes than it was in normal eyes.

## 1. Introduction

The pars plana vitrectomy (PPV) is a common procedure for the treatment of vitreoretinal diseases including vitreous hemorrhage, retinal detachment, epiretinal membrane, and macular hole. With the recent introduction of a sutureless vitrectomy technique, vitreoretinal surgeons are now performing vitrectomies more routinely and with fewer serious complications [1, 2]. It has been well established that the intraocular pressure (IOP) increases immediately after a vitrectomy [3–6]. The mechanism of this increased IOP is unclear, although it responds well to conventional ocular hypotensive medications [3, 4]. Although several studies have reported that a vitrectomy may induce glaucoma long after surgery, the association remains unclear [3, 7, 8]. According to Yu et al., neither the incidence of late-onset open-angle glaucoma (OAG) nor that of ocular hypertension (OHT) increases in patients after a vitrectomy [9]. Diurnal IOP change and peak IOP are important factors in glaucoma development and they are essential for evaluating the precise state of the IOP [10–12]. The studies mentioned above did not investigate the diurnal IOP pattern and its variation, and these patterns may provide a crucial link between the vitrectomy and development of glaucoma. In this study, we analyzed the effect of vitrectomy on the diurnal change of IOP.

## 2. Methods

From May to December 2007, we recruited patients who were admitted for cataract surgery and who had undergone a previous vitrectomy. All participants underwent full ophthalmologic examinations before admission into the study. This exam included slit biomicroscopy, Zeiss four-mirror indentation gonioscopy, dilated fundus indirect biomicroscopy, Snellen best corrected visual acuity, specular biomicroscopy, and optical biometry (IOL-Master, Carl Zeiss). Patient history was evaluated, including other systemic and ocular diseases, current medications, and prior surgeries. We obtained the patients' informed consent after they were admitted for cataract surgery. An experienced ophthalmologist used a Goldman applanation tonometer to measure the diurnal IOP every two hours between 9 a.m. and 11 p.m..

We compared the diurnal IOP data in patients who had one vitrectomized eye with a normal fellow eye and with age-, gender-, and axial length-matched controls. A vitrectomized eye was defined according to the following criteria: (1) the eye had undergone one successful PPV by a retinal specialist without any complications, (2) a phakic eye had an open angle without peripheral anterior synechiae (PAS), and (3) the eye was not exposed to antiglaucoma eye drops for at least four months prior to the study. To avoid the influence of tamponade foreign material on IOP, we excluded eyes with a history of silicone injection or eyes that had undergone surgery within the last four months. A patient was defined to have a normal fellow eye by the following criteria: (1) the eye had an open angle without PAS and had never been operated on, (2) the eye had not been exposed to antiglaucoma eye drops for at least four months prior to the study, and (3) the eye did not have any pathology or abnormality associated with IOP on full ophthalmologic examination.

The inclusion criteria for normal control eyes were the same as those for normal fellow eyes as described above. Only the right eyes of control individuals were included. Patients were excluded from analysis if they had a history of ocular surgery (not including vitrectomy), use of antiglaucoma medication, PAS > 90 degrees as measured with gonioscopy, inflammation, or anterior chamber reaction.

We compared the diurnal IOP and ocular biometries of vitrectomized eyes to their normal fellow eyes and to control eyes. To minimize selection bias, two separate ophthalmologists measured IOP and the data independently and in parallel. Both ophthalmologists were blinded to patient information.

Paired *t*-tests were conducted using SPSS 18.0 (Chicago, IL, USA) to analyze the difference in IOP between vitrectomized and normal eyes.

## 3. Results

This study included 21 individuals (12 males and 9 females) with one vitrectomized eye and one normal eye and 21 normal control individuals. The mean patient age was 57.52 ± 12.80 years (range: 21–78 years) and 17 (81.0%) of the vitrectomized eyes were right eyes. The preoperative indications for vitrectomy included vitreous hemorrhage (9 eyes, 42.9%), retinal detachment (5 eyes, 23.8%), epiretinal membrane (4 eyes, 19.0%), macular hole (2 eyes, 9.5%), and endophthalmitis (1 eye, 4.8%). After the vitrectomy, gas was injected into 11 eyes (C3F8 in 8 eyes and SF6 in 3 eyes), and the other eyes were not injected with any tamponade material (10 eyes, 47.6%). Mean IOPs were 13.33 ± 2.90 mmHg in the eyes with C3F8 gas tamponade, 13.07 ± 3.35 mmHg in the eyes with SF6 gas tamponade, and 13.61 ± 2.88 mmHg in the eyes without tamponade. The mean interval time between surgery and IOP measurement was 36.12 ± 39.20 months (range: 4–140 months) (Table 1).

There were no strong correlations between the IOP of vitrectomized eyes and those of normal fellow eyes. Also, the correlation coefficient between the IOP of the vitrectomized eye and that of the normal fellow eye was low in the morning when the IOP was relatively high. The mean correlation coefficients of the IOPs between bilateral eyes are summarized in Table 2.

Comparisons of IOP and other ocular parameters between vitrectomized eyes and normal fellow eyes are described in Table 3. There were no significant differences between the diurnal measurements, the average IOP, the minimum IOP, the maximum IOP, or the IOP fluctuation (calculated as [maximum IOP − minimum IOP]) between vitrectomized and normal fellow eyes. Other ocular parameters including endothelial cell count, corneal pachymetry, axial length, anterior chamber depth, and keratometry also were not significantly different between the two groups (Table 3).

The comparisons of IOP and ocular parameters between vitrectomized eyes and age-, gender-, and axial length-matched control eyes are described in Table 4. There were no significant differences in the diurnal measurement, average IOP values, minimum IOP values, or maximum IOP values between the two groups. However, there was significantly greater IOP fluctuation in vitrectomized eyes (3.38 ± 3.54 mmHg) compared to the control eyes (1.65 ± 0.95 mmHg, *P* = 0.031). No other ocular parameters were significantly different between the two groups (Table 4).

**Figure 1:**
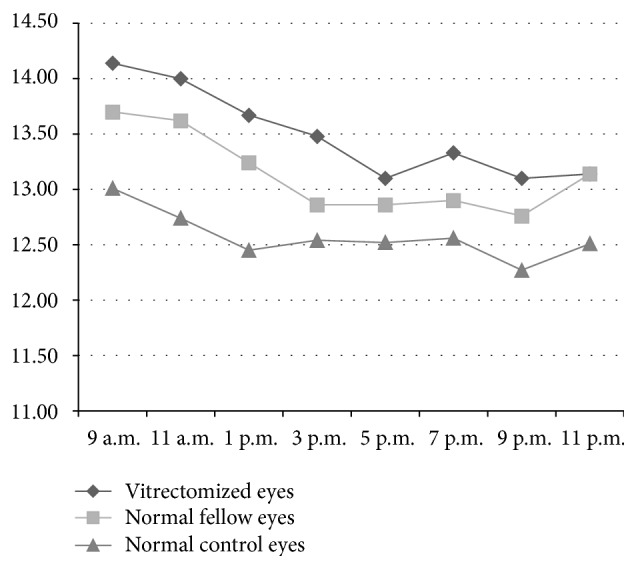
Diurnal intraocular pressure of vitrectomized eyes, normal fellow eyes, and normal control eyes. There was no significant difference in diurnal IOP among the three groups.

Following a typical diurnal pattern, the IOP in all subjects was the highest in the morning and gradually decreased over the course of the day (Figure 1).

## 4. Discussion

In this study, we found that the IOP profiles were not significantly different in vitrectomized eyes compared with that of normal eyes except for fluctuations in the diurnal IOP. The IOP diurnal fluctuation was larger in vitrectomized eyes than it was in age-, gender-, and axial length-matched controls' normal eyes. Furthermore, although it was not statistically significant, the IOP diurnal fluctuation was larger in patients' vitrectomized eyes compared to their normal fellow eyes.

It is still controversial whether or not a vitrectomy affects the IOP in the development of postoperative glaucoma. No prior studies have evaluated the diurnal IOP. However, there are contradictory results from prior studies with regard to the association between vitrectomies and IOP. Yu et al. [9] demonstrated that a vitrectomy did not increase the risk of glaucoma or OHT in 441 vitrectomized patients. They found that OHT and OAG occurred in 4.31% of vitrectomized eyes; 2.49% of the control eyes were afflicted with OHT and 2.95% of control eyes experienced OAG. Although a larger proportion of OHT and OAG occurred in vitrectomized eyes, there was no statistically significant difference between the two groups.

In contrast, Fujikawa et al. reported that the mean IOP increased after a vitrectomy in patients with macular holes [13]. Similarly, Ki-I et al. reported that although the immediate change in the IOP after a phacovitrectomy was minimal, some eyes developed increased IOP after several months [14]. More recently, Wu et al. reviewed the IOP of 198 patients who underwent a vitrectomy and demonstrated that the incidence of high IOP (≥24 mmHg) or increased IOP (≥5 mmHg) was significantly greater in vitrectomized eyes than in control eyes [15].

There are several possible mechanisms by which the IOP increases soon after a vitrectomy. For example, IOP can increase as a result of surgical complications including hemorrhage, inflammation, and gas tamponade [4, 16–18]. This temporary IOP elevation can be easily controlled with antiglaucoma eye drops [3, 4]. In other cases, late-onset glaucoma may develop [17, 19]. Given this risk, it is very important to identify any long-term harmful effects that vitrectomy has on the eye.

There are many reports regarding IOP and the prevalence of OHT or OAG after a vitrectomy. However, none of these studies reported the diurnal IOP. Therefore, the association between a vitrectomy and diurnal IOP had not been previously characterized. It has been well established that there is a diurnal variation in IOP and it may be an important factor in glaucoma development [12]. IOP is not a fixed value and it varies even over short-term periods. Some studies have suggested that the diurnal fluctuation may be increased in glaucoma or OHT patients [10–12, 20]. The circadian rhythm of ocular perfusion pressure could contribute to the mechanism of changing diurnal fluctuations [21], though the mechanism is not clearly understood. Regardless, it is important to study diurnal IOP patterns in order to understand the variation in IOP and characterize pathological IOP patterns. We investigated the diurnal IOP to determine whether it is affected by a vitrectomy.

In this study, we compared the diurnal IOP of vitrectomized eyes with normal fellow eyes. There were no significant differences between vitrectomized eyes and normal fellow eyes in the diurnal IOP, minimum IOP, maximum IOP, or fluctuations of IOP.

One retrospective study of OAG patients suggested that daytime peak IOP might be important in predicting the progression of glaucoma [22]. Konstas et al. reported that patients with a daytime peak IOP of 18 mmHg or lower (readings at 10 a.m., 2 p.m., and 6 p.m.) generally did not experience worsening of their glaucoma. We measured IOP with a shorter interval in between readings (2 hours), and there were no significant differences in the peak IOP between vitrectomized eyes and normal eyes.

We also compared the diurnal IOP of the vitrectomized eye with age-, gender-, and axial length-matched control eyes to eliminate the effects of axial length on the IOP [23]. Similarly, there were no significant differences in terms of diurnal IOP, minimum IOP, or maximum IOP values between vitrectomized eyes and control eyes. However, the diurnal IOP fluctuations were wider in the vitrectomized eyes than they were in the control eyes.

We did not find a strong correlation in the IOP between vitrectomized eyes and normal fellow eyes. In a previous study, the correlation between right and left eyes in normal subjects almost exceeded 0.8 [23]. However in our study, there were relatively low correlations between vitrectomized eyes and normal fellow eyes.

Even if there are no major complications after the vitrectomy, the IOP may change. There are several possible mechanisms by which the IOP changes after vitrectomy. Chang reported that increased oxidative stress on the trabecular meshwork after a vitrectomy might increase the IOP and the risk of glaucoma in the long term [7]. Moreover, Fujikawa et al. suggested that inflammatory stress or the physical effects of the vitrectomy could be the cause of increased IOP [13]. Although the mechanism is unclear, various effects of a vitrectomy can influence diurnal changes of the IOP.

Our study has several limitations. For one, cross-sectional studies have inherent limitations. We also used a small sample size and did not control for the preoperative diagnosis. In addition, because patients who had used antiglaucoma eye drops were excluded, the IOP changes after the vitrectomy may have been underestimated. A well-designed, longitudinal, prospective study is necessary to further evaluate IOP in vitrectomized eyes.

In conclusion, a vitrectomy may change diurnal IOP in the long term. However, the IOP changes observed herein were within an acceptable range. There were no severe complications associated with increased IOP after a vitrectomy. Considering the importance of the IOP diurnal variation, these data help to describe the status of the eye after a vitrectomy.

## Figures and Tables

**Table 1:** Demographics and basic patient data of the participants who underwent a vitrectomy and the age-, gender-, and axial length-matched controls.

Characteristics	Vitrectomized eyes (*n* = 21)	Control eyes (*n* = 21)	*P* value
Age (years)	57.52 ± 12.80 (21–78)	57.52 ± 12.80 (21–78)	
Eyes			
OD	17 (81.0%)	17 (81.0%)	
OS	4 (19.0%)	4 (19.0%)	
Gender			
Male	12 (57.1%)	12 (57.1%)	
Female	9 (42.9%)	9 (42.9%)	
Hypertension	10 (47.6%)	9 (42.9%)	0.283^a^
Diabetes	9 (42.9%)	6 (28.6%)	0.328^a^
Systolic blood pressure (mmHg)	127.67 ± 16.55 (110–170)	118.76 ± 10.83 (110–140)	0.098^b^
Diastolic blood pressure (mmHg)	76.91 ± 9.55 (60–98)	75.14 ± 11.30 (50–90)	0.603^b^
Time elapsed since surgery (months)	36.12 ± 39.20 (4–140)		
Reason for vitrectomy			
Vitreous hemorrhage	9 (42.9%)		
Diabetic retinopathy	6 (28.6%)		
Retinal vein obstruction	2 (9.5%)		
Trauma	1 (4.8%)		
Retinal detachment	5 (23.8%)		
Epiretinal membrane	4 (19.0%)		
Macular hole	2 (9.5%)		
Endophthalmitis	1 (4.8%)		
Tamponade			
None	10 (47.6%)		
C3F8	8 (38.1%)		
SF6	3 (14.3%)		

^a^Chi-square test.

^b^Independent sample *t*-test.

**Table 2:** Correlation of mean intraocular pressure (mmHg) between vitrectomized eyes and fellow eyes.

Time	Vitrectomized eye	Normal fellow eye	*r* ^2^
9 a.m.	14.14 ± 5.15	13.71 ± 2.92	0.391
11 a.m.	14.00 ± 3.54	13.62 ± 2.75	0.519
1 p.m.	13.67 ± 3.45	13.24 ± 2.79	0.652
3 p.m.	13.48 ± 3.72	12.86 ± 3.05	0.708
5 p.m.	13.10 ± 3.49	12.86 ± 2.73	0.736
7 p.m.	13.33 ± 3.68	12.90 ± 2.98	0.763
9 p.m.	13.10 ± 3.73	12.76 ± 2.91	0.860
11 p.m.	13.14 ± 3.85	13.14 ± 3.21	0.845

**Table 3:** Comparing the diurnal intraocular pressure (IOP) and ocular parameters between vitrectomized eyes and normal fellow eyes.

	Vitrectomized eye	Normal fellow eye	*P* value^a^
IOP (mmHg)			
9 a.m.	14.14 ± 5.15	13.71 ± 2.92	0.631
11 a.m.	14.00 ± 3.54	13.62 ± 2.75	0.486
1 p.m.	13.67 ± 3.45	13.24 ± 2.79	0.347
3 p.m.	13.48 ± 3.72	12.86 ± 3.05	0.174
5 p.m.	13.10 ± 3.49	12.86 ± 2.73	0.554
7 p.m.	13.33 ± 3.68	12.90 ± 2.98	0.289
9 p.m.	13.10 ± 3.73	12.76 ± 2.91	0.319
11 p.m.	13.14 ± 3.85	13.14 ± 3.21	1.000
Average	13.43 ± 3.55	13.13 ± 2.79	0.441
Minimum	11.86 ± 3.71	12.00 ± 2.95	0.651
Maximum	15.24 ± 4.68	14.29 ± 2.72	0.272
Fluctuation	3.38 ± 3.54	2.29 ± 1.23	0.135
Ocular parameters			
Endothelial cell count	2490.67 ± 427.56	2515.43 ± 395.70	0.807
Pachymetry (*μ*m)	581.00 ± 42.54	577.00 ± 46.36	0.532
Axial length (mm)	23.86 ± 1.43	23.75 ± 1.36	0.544
Anterior chamber depth (mm)	3.36 ± 0.48	3.31 ± 0.42	0.594
Keratometry (D)	44.05 ± 1.52	44.12 ± 1.41	0.480

^a^Paired sample *t*-test.

**Table 4:** Comparing the diurnal intraocular pressure and ocular parameters between the vitrectomized eyes and the age-, gender-, and axial length-matched normal controls eyes.

	Vitrectomized eye	Normal control eye	*P* value^a^
IOP (mmHg)			
9 a.m.	14.14 ± 5.15	13.01 ± 2.30	0.289
11 a.m.	14.00 ± 3.54	12.74 ± 2.67	0.180
1 p.m.	13.67 ± 3.45	12.45 ± 2.62	0.188
3 p.m.	13.48 ± 3.72	12.54 ± 2.67	0.298
5 p.m.	13.10 ± 3.49	12.52 ± 2.41	0.508
7 p.m.	13.33 ± 3.68	12.56 ± 2.45	0.380
9 p.m.	13.10 ± 3.73	12.27 ± 2.56	0.376
11 p.m.	13.14 ± 3.85	12.51 ± 2.77	0.522
Average	13.43 ± 3.55	12.62 ± 2.44	0.352
Minimum	11.86 ± 3.71	11.83 ± 2.56	0.973
Maximum	15.24 ± 4.68	13.48 ± 2.32	0.108
Fluctuation	3.38 ± 3.54	1.65 ± 0.95	0.031^a^
Ocular parameters			
Pachymeter (*μ*m)	581.00 ± 42.54	557.23 ± 38.57	0.075
Axial length (mm)	23.86 ± 1.43	23.83 ± 1.19	0.909
Anterior chamber depth (mm)	3.36 ± 0.48	3.12 ± 0.40	0.118

^a^Paired sample *t*-test.
